# Cuticular Hydrocarbon Profile of Parasitic Beetles, *Aethina tumida* (Coleoptera: Nitidulidae)

**DOI:** 10.3390/insects12080751

**Published:** 2021-08-19

**Authors:** Anna Papach, Federico Cappa, Rita Cervo, Leonardo Dapporto, Rammohan Balusu, Geoffrey R. Williams, Peter Neumann

**Affiliations:** 1 Vetsuisse Faculty, Institute of Bee Health, University of Bern, 3003 Bern, Switzerland; peter.neumann@vetsuisse.unibe.ch; 2 Agroscope, Swiss Bee Research Centre, 3097 Bern, Switzerland; 3 Dipartimento di Biologia, Università di Firenze, Via Madonna del Piano 6, Sesto Fiorentino, 50019 Firenze, Italy; rita.cervo@unifi.it (R.C.); leondap@gmail.com (L.D.); 4 Department of Entomology & Plant Pathology, Auburn University, Auburn, AL 36849, USA; balusrr@auburn.edu (R.B.); williams@auburn.edu (G.R.W.)

**Keywords:** *Aethina tumida*, *Apis mellifera*, chemical profile, honey bee, nestmate recognition, parasite

## Abstract

**Simple Summary:**

Social insects use cuticular hydrocarbons for chemical recognition and communication. Cuticular hydrocarbons can also be exploited by parasites to their advantage for undermining host recognition systems. The small hive beetle (SHB) is a parasite of honey bee colonies but can also infest nests of other bee species. However, its chemical profile is still not known. For the first time, the present study investigated the SHB chemical profile and compared it with that of its honey bee host. The results show that the SHB has a low chemical profile that is similar to its honey bee host’s. However, while honey bees had a clear colony-specific chemical profile, SHBs did not. The generic chemical profile of the SHB is most likely linked to its free-flying behaviour in the field as these parasites are known to switch between host colonies, possibly limiting the acquisition of a colony specific chemical profile. Our findings also suggest that SHBs do not exploit any finely tuned chemical strategy to conceal their presence inside host colonies and probably rely on behavioural adaptations.

**Abstract:**

Cuticular hydrocarbons (CHCs) cover insects’ bodies and play important roles in chemical communication, including nestmate recognition, for social insects. To enter colonies of a social host species, parasites may acquire host-specific CHCs or covertly maintain their own CHC profile by lowering its quantity. However, the chemical profile of small hive beetles (SHBs), *Aethina tumida*, which are parasites of honey bee, *Apis mellifera*, colonies, and other bee nests, is currently unknown. Here, adults of SHB and honey bee host workers were collected from the same field colonies and their CHC profiles were analysed using GC-MS. The chemical profiles of field-sampled SHBs were also compared with those of host-naive beetles reared in the laboratory. Laboratory-reared SHBs differed in their CHC profiles from field-sampled ones, which showed a more similar, but ten-fold lower, generic host CHC profile compared to host workers. While the data confirm colony-specific CHCs of honey bee workers, the profile of field-collected SHBs was not colony-specific. Adult SHBs often commute between different host colonies, thereby possibly preventing the acquisition of a colony-specific CHC profiles. An ester was exclusive to both groups of SHBs and might constitute an intraspecific recognition cue. Our data suggest that SHBs do not use any finely tuned chemical strategy to conceal their presence inside host colonies and instead probably rely on their hard exoskeleton and defence behaviours.

## 1. Introduction

Discrimination between group members and foreign individuals represents a key feature of any social species. In social insect colonies, the ability to recognize colony members (i.e., nestmates) is essential to maintain group integrity, avoid the exploitation of colony resources, and defend the colony from parasites and pathogens [1,2,3]. Although different sensory channels can be involved in the recognition process, depending on the species and context (e.g., stage of the colony) [4,5,6], such differentiation is primarily governed by odour cues in which cuticular hydrocarbons (CHCs) covering the body surface of individual insects are of particular significance [7]. Serving as a basis for nestmate recognition, CHCs are usually qualitatively similar among individuals of a species but can vary in their relative amounts among individuals of colonies of the same species [8,9,10].

Many parasites have developed a number of strategies to deceive and exploit this recognition system, and to gain access to valuable colony resources [11,12,13,14]. They can employ chemical mimicry so that their chemical profile matches that of their host, or they can adopt a strategy of chemical insignificance or neutral odour by reducing the quantity of chemical cues or selectively suppressing the expression of those cues that are important for recognition [15]. This decreases the chances it will be detected by the host. To date, only a few studies have addressed the question of quantitative perception thresholds for recognition, and demonstrated that in practice [16,17,18]. Parasites often employ more than one strategy to overcome the host recognition system. For example, they may change their CHCs depending on the progression of the invasion, such as the butterfly *Maculinea rebeli* which synthesises host-specific compounds before invading the nest of its ant host *Myrmica schenki*. Once inside the colony, it fine-tunes its chemical profile to the host’s colony odour by acquiring compounds, possibly through trophallaxis with the host [19].

Insects obtain most of their CHCs through synthesis that starts during the larval stage and slows down during the post-feeding stage; however, it will usually take several days after emergence to develop a complete CHC profile [8,20,21]. Another way to acquire CHCs is through contacts with nest material [22,23,24]. Furthermore, CHCs can be transferred between conspecifics via direct interactions such as grooming, body contact, and trophallactic exchange [25,26]. The CHC profile can also change with individual age or health condition [27,28,29], as well as nutritional status [30,31].

The small hive beetle (SHB), *Aethina tumida*, is a parasite of honey bee, *Apis mellifera*, colonies native to sub-Saharan Africa [32]. In 1996, it was first reported in the USA and started its global journey reaching all of the continents except Antarctica [33,34,35]. Within its native range, it is usually considered to be a minor pest [36]; however, it can have a considerable impact on honey bee colonies in its invaded ranges [35]. SHB can also infest colonies of other social bees, as well as solitary bee nests [35,37,38], but the role of these alternative hosts is poorly understood. Larval and adult SHBs feed on honey, pollen, host brood, dead or live adult bees, and can even trick honey bees into trophallactic feeding [39]. Adult SHBs are known to conduct long-range dispersal flights searching for a host colony to enter [40]. Within the apiary, SHBs usually have a non-random distribution, tending to aggregate in particular colonies [41,42], but they can also frequently move among colonies within an apiary [43]. It has been observed that honey bees are usually less aggressive towards adult SHBs that have been inside their colonies compared to newly introduced ones [44]. However, the chemical profile of the SHB and its potential role in overcoming the host defence is still not known.

Here, we characterized for the first time the chemical profile of the adult SHB and investigated the similarities with its honey bee host profile. Based on previous observations of lower aggressiveness exhibited by honey bees towards nestmate SHBs [44], and the occurrence of trophallactic feeding between honey bees and SHBs [39], we hypothesized that after entering the host colony, SHB could express a colony-specific profile similar to its host. To test this, the CHC profile of the SHB and honey bee workers from the same colonies were characterized and compared. Additionally, we analysed the chemical profile of laboratory-reared SHBs naive to honey bees to understand if SHBs already show a characteristic chemical signature prior to entering a host colony.

## 2. Materials and Methods

### 2.1. Experimental Design

Adult SHBs (N = 48 in total, 8–10/colony) and adult honey bee workers (N = 48 in total, 9–10/colony) were collected from five different queenright honey bee, *A. mellifera*, colonies in three different apiaries around Auburn, AL, USA in Summer 2019. Honey bee workers were collected from brood frames and SHB from entire hives of local naturally honey bee colonies using aspirators [45]. Experimental insects were freeze-killed, stored at −20 °C, and then used for the subsequent chemical analysis.

To obtain laboratory-reared bee naive individuals, SHB adults were collected from naturally infested honey bee colonies and used to initiate laboratory rearing following standard protocols [45]. In brief, freshly hatched SHB larvae were fed by providing them with a honey bee worker brood frame until they had reached the post-feeding wandering stage and then transferred into a 473 mL glass jar filled with suitable autoclaved soil for pupation. Pupation containers were kept at 25 °C, 80% RH, 24 h dark until adult emergence [46]. Upon emergence, adult SHBs [N = 10] were kept in incubators with sugar water [45] for seven days and then freeze killed for further chemical analysis. Samples were stored at −20 °C.

CHC extracts were obtained by washing each honey bee worker in 1 mL of hexane and each SHB in 0.5 mL of hexane for 15 min. The different amounts of solvents were reflecting differences in body size. Then, the extracts were allowed to evaporate and dried samples were covered with foil and transported to Italy for coupled gas–chromatography mass spectrometry analysis (GS-MS).

### 2.2. GC-MS Analyses

Dried extracts of all specimens (N = 9 laboratory-reared SHBs; N = 47 field-collected SHBs; N = 47 honey bee workers) were re-suspended in 100 μL of pentane and transferred to a conical glass insert inside the original vial used for extraction. The solvent was then dried under a stream of nitrogen and the SHB samples were re-suspended in 20 μL of heptane with 70 ng/μL of heptadecane (n-C17) as the internal standard. For the honey bee worker samples, 80 μL of heptane with 70 ng/μL of heptadecane (n-C17) as internal standard. The final volume of resuspension was quadrupled for honey bee samples since preliminary analyses of a few specimens showed peak saturation for honey bee extracts re-suspended in 20 μL of heptane added with heptadecane due to the high quantity of CHCs extracted from honey bee workers. One μL of the extract was injected in a Hewlett Packard (Palo Alto, CA, USA) 5890A gas chromatograph (GC) coupled to an HP 5970 mass selective detector (using 70 eV electronic ionization source). A fused ZB-WAX-PLUS (Zebron) silica capillary column (60 m × 0.25 mm × 0.25 mm) was installed in the GC. The injector port and transfer line temperatures were set at 200 °C and the carrier gas was helium (at 20 PSI head pressure). The temperature protocol was from 50 °C to 320 °C at a rate of 10 °C/min, and the final temperature was kept for 5 min. Injections were performed in splitless mode (1 min purge valve off). Data acquisition and analysis were performed using the Chem Station G1701 BA (version B.01.00)—Copyright© Hewlett-Packard 1989–1998. Compounds corresponding to different peaks in each chromatogram were identified on the basis of their retention time and mass spectra. Mass spectra were compared with mass spectral electronic libraries (Wiley 275, NIST 2.0).

### 2.3. Statistical Analyses

The amount of each compound was evaluated by dividing its abundance by the abundance of n-C17 (multiplied by 4 for honey bee workers because of the higher dilution). The resulting amount was transformed by the method provided by Aitchison [47], which avoids bias due to the use of compositional data in multivariate analyses:

Zij = lnYij/g(Yj)

where Yij is the amount of peak i for individual j, g(Yj) is the geometric mean of the amounts of all peaks for individual j, and Zij is the transformed amount of peak i for individual j.

Univariate and multivariate analyses were applied to compare the possibility of attributing honey bees and SHBs to the colony they belong to based on chemical composition. With this aim, first was performed a Partial Least Square Discriminant Analysis (PLSDA) as implemented in the mixOmics R package [48]. As a grouping variable, we used eleven groups identified by different species (SHBs vs. honey bees), colony membership, and SHBs laboratory or field collection status as a priori grouping variable. The composition for all compounds was compared among laboratory-reared SHBs, field-collected SHBs, and honey bees by using Kruskal–Wallis test paired with post hoc comparisons (kruskal.test and pairwise.wilcoxon.test of the stats R package). *p* values from multiple Kruskal–Wallis tests were adjusted by using the Benjamini and Hochberg procedure implemented in the p.adjust function of the stat R package. To detect if SHB changed its profile after entering a honey bee colony to match that of the host chemical, we calculated chemical dissimilarity among the signature centroid of honey bee workers (average transformed amount of each compound), host-naive SHBs reared in the laboratory, and SHBs collected from host colonies in the field. Chemical dissimilarity was calculated using the Bray–Curtis dissimilarity (vegdist function of the vegan R package). Dissimilarities to honey bee worker centroid to laboratory-reared and field-collected SHBs were compared with a Mann–Whitney test.

The possibility to attribute honey bee workers to their maternal colonies based on the typical colony profiles was tested by a jackknife procedure where a sparse PLSDA (SPLSDA), more conservative than a PLSDA since it allows the inclusion of a reduced number of variables per each discriminant component (ten variables in our assessment), was performed on all the specimens but one using colony membership as a grouping variable. Then, colony membership of the excluded specimen was predicted on the basis of their CHCs composition. Colony membership of field-collected SHBs was predicted on the full SPLSDA model obtained for honey bee workers. The percentage of correctly attributed cases was used as a measure of the possibility to blindly attribute individuals to their colonies. Finally, the overall quantity of compounds (not transformed by Aitchison formula) was compared among laboratory-reared SHBs, field-collected SHBs, and honey bee workers by using a Kruskal–Wallis test with post hoc comparisons. All calculations were performed using the program R [49].

## 3. Results

**Figure 1:**
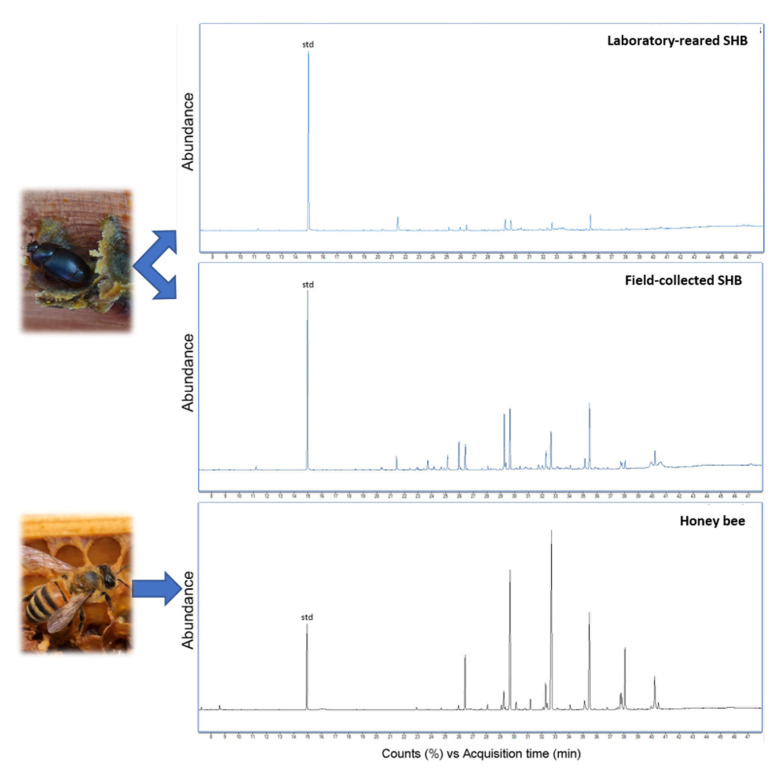
Representative chromatograms of laboratory-reared and field-collected small hive beetles, *Aethina tumida* (SHB), and their honey bee host workers, *Apis mellifera*. Abundance of compounds is shown over Counts (%) vs. Acquisition time (min).

Laboratory-reared and field-collected SHBs had a low chemical profile that was similar to its honey bee host (Figure 1). Laboratory-reared SHBs had a less pronounced chemical profile than SHBs collected from the field.

### 3.1. Colony Membership Allocation

**Figure 2:**
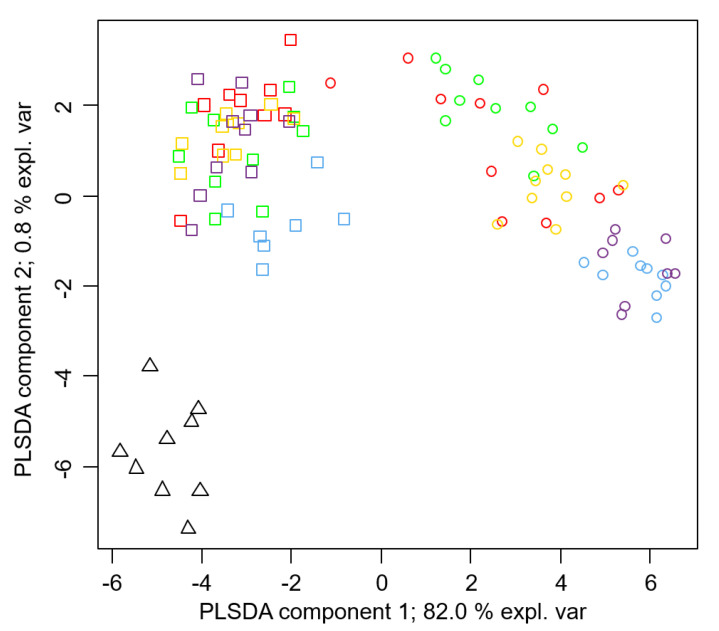
A scatterplot of first and second discriminant components distinguishing chemical profiles with respect to 11 groups differentiated based on species (either small hive beetles, *Aethina tumida* (SHB), or honey bee workers, *Apis mellifera*), rearing condition, and colony membership (triangles-laboratory-reared SHBs (1 group); squares-field-collected SHBs (5 groups); circles-honey bee workers (5 groups)). Different colours indicate colony membership for both honey bees and field-collected SHBs. Partial Least Square Discriminant Analysis (PLSDA).

A Partial Least Square Discriminant Analysis (PLSDA) separating 11 groups of honey bees and SHBs (five colonies for honey bees, five colonies for field-collected SHBs, and one group of laboratory-reared SHBs) based on their CHC signatures showed that the laboratory-reared SHBs, field-collected SHBs, and honey bees formed three distinct clusters (Figure 2). Most of the variation was due to the first component, thereby explaining 82.0% of the chemical variation and separating the two species. A much lower variance was explained by the second component (0.8%), encompassing differences among laboratory-reared and field-collected SHBs and honey bees from different colonies. The importance of compounds in the PLSDA solution was reported as loadings for the first two components in Table 1.

Field-collected SHBs showed changes in chemical composition compared to laboratory-reared ones, thereby resembling a more generic honey bee profile. Indeed, a jackknife procedure performed to blindly attribute each honey bee sample to a colony based on comparing each chemical profile with a SPLSDA model constructed on all other honey bees showed that 68.1% of honey bees were correctly classified. Conversely, when profiles of SHBs were attributed to a colony based on SPLSDA models constructed on honey bee profiles, only 14.6% of individuals were correctly classified. Accordingly, a the PLSDA where honey bees and field-collected SHBs were grouped to their colony membership showed that honey bee workers form distinct groups based on the two first discriminant components alone (Figure 2); conversely, field-collected SHB individuals were largely admixed among colonies, which denoted no chemical characterization (Figure 2).

**Figure 3:**
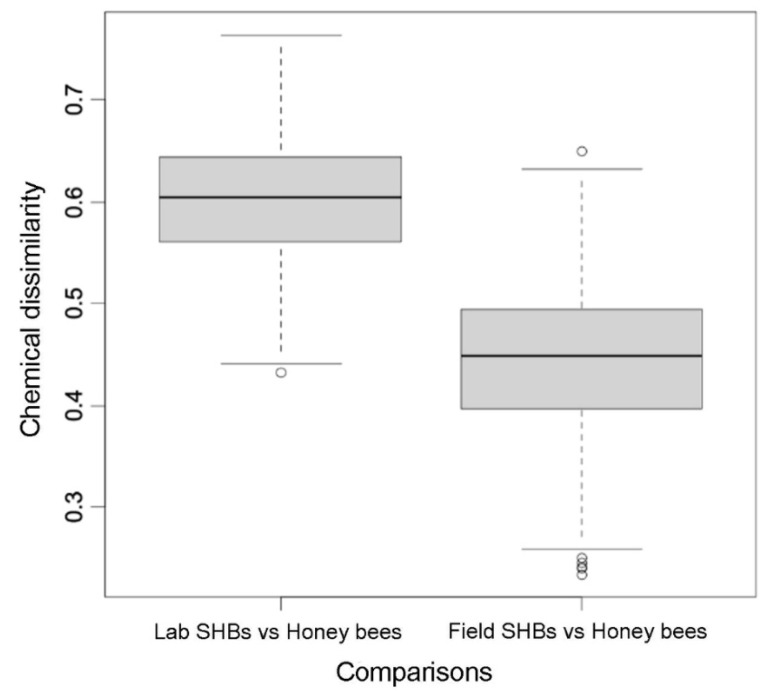
Cuticular hydrocarbons dissimilarities between laboratory-reared small hive beetles (Lab SHBs), *Aethina tumida*, and the field-collected honey bee, *Apis mellifera*, workers and between field-collected SHBs (Field SHBs) and honey bee workers. Medians and 25% quartiles are shown.

Chemical dissimilarity, calculated as the pairwise Bray–Curtis dissimilarity from the chemical signature centroid of field-collected honey bee workers, was significantly higher for the CHC profiles of laboratory-reared SHBs than for field-collected SHBs (Mann–Whitney test; W = 411, *p* < 0.001, Figure 3).

### 3.2. Qualitative Chemical Analysis

Many compounds contributed to the observed pattern as it can also be observed in univariate comparisons, where 52 compounds of 61 showed an overall significant difference among groups (Table 1). In pairwise comparisons, 17 compounds differed between laboratory and field-collected SHBs, 44 between laboratory-reared SHBs and field-collected honey bee workers, as well as 40 between field-collected SHBs and honey bee workers. The chemical profile of laboratory-reared SHBs showed the lowest number of detected compounds (31 out of 61, Table 1); 54 different compounds were identified in field-collected SHBs, while only one peak corresponding to an unidentified ester (putatively acetic acid n-octadecyl ester) was exclusive to both groups of SHBs and not found in any honey bee worker. The majority of compounds differing between laboratory-reared and field-collected SHBs consisted of alkenes and methyl-branched alkanes (15 compounds out of 17, Table 1), which were absent or less abundant in laboratory-reared SHBs, apart from me-C_27a,_ me-C_28,_ me-C_29b_, and me-C_31a_ which were present in a lower amount or not found in field-collected SHBs (Table 1).

### 3.3. Quantitative Chemical Analyses

**Figure 4:**
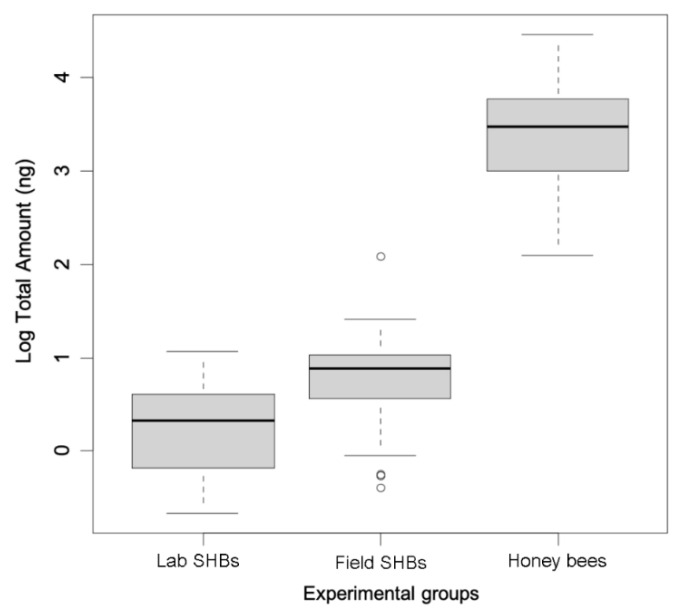
Overall amounts of cuticular hydrocarbon compounds between laboratory-reared small hive beetles (Lab SHBs), *Aethina tumida*, field-collected SHBs (Field SHBs), and field-collected honey bee workers, *Apis mellifera*. Medians and 25% quartiles are shown.

The overall amount of chemicals significantly differed between laboratory-reared SHBs, field-collected SHBs, and honey bee workers (Kruskal–Wallis test chi–squared = 78.90, *p* < 0.001). Post hoc comparisons showed a significant effect in all pairwise comparisons (pairwise Wilcoxon test: laboratory-reared SHBs vs. field-collected SHBs, *p* < 0.005; laboratory-reared SHBs vs. honey bee workers, *p* < 0.0001; field-collected SHBs vs. honey bee workers, *p* < 0.0001; Figure 4). The three groups also differed in the total amount of CHCs calculated through the 70 ng/μL of heptadecane (n-C17) as internal standard (total CHCs amount: laboratory-reared SHBs, 1.98 ± 1.02 μg; field-collected SHBs, 3.28 ± 1.67 μg; honey bee workers, 48.42 ± 25.48 μg; Kruskal–Wallis test chi−squared = 79.29, *p* < 0.0001, post hocs: laboratory-reared SHBs vs. field-collected SHBs, *p* = 0.008; laboratory-reared SHBs vs. honey bee workers, *p* < 0.0001; field-collected SHBs vs. honey bee workers, *p* < 0.0001).

## 4. Discussion

Our data provided the first characterization of adult SHB CHC profiles. Laboratory-reared SHBs had a less pronounced CHC profile both in terms of quantity and chemical composition compared to field-collected ones, which displayed a low generic host CHC profile. However, while the data confirmed colony-specific CHC profiles of honey bee workers [8,10], SHBs did not display such host colony-specific profiles.

With the exception of a single ester, cuticular profiles of adult SHBs and honey bee workers shared all compounds. The absence of an evident colony signature in field-collected SHBs suggests that these parasites do not use finely tuned chemical mimicry to conceal their presence inside a honey bee host colony. It is likely that the observed generic CHC host profile of adult SHBs may be linked to the behaviour of free-flying adults in the field. Indeed, it has been previously reported that adult SHBs can easily move among honey bee colonies of the same apiary and even disperse to distant apiaries [40,42,43,50], thereby possibly limiting the acquisition of a host colony-specific CHC profile. Even though SHBs appear not to mimic a colony-specific signature, a hard exoskeleton and various defence behaviours [35] are apparently sufficient to survive inside host colonies. An example is the turtle defence posture, where the SHB tucks its head under the pronotum, presses legs and antennae tightly to the body and stays motionless [51]. Indeed, usually less than one percent of honey bee worker attacks result in bees grabbing an SHB antenna [39], and the killing of adult SHBs by honey bee workers is extremely rare (PN unpublished observation). Moreover, SHB can also infest nests of bumble bees, stingless bees, and solitary bees [35,37]. In light of such a broad potential host spectrum and the mobile nature of free-flying adult SHBs, host colony-specific CHC profiles might be costly.

The generic host chemical profile observed in field-collected SHBs did not seem to be immediately developed by SHBs after emergence, since a noticeable increase in the total amount of CHCs and in the number of compounds was observed in beetles collected from host colonies. The higher complexity of CHCs in field-collected SHBs might be due to age [52,53,54]. However, the adult SHBs were kept for one week after emergence under controlled laboratory conditions, which was sufficient to develop a full CHC profile in other insect species [20,55]. Furthermore, in field-collected SHBs there was no increase in the amount of compounds that were already present in laboratory-reared ones as expected in case of an age-related CHC increase [50]. Instead, there was a consistent rise in both the total amount of CHCs and the number of compounds, which almost doubled in field-sampled SHBs. Since the cuticular compound dynamics in insects can depend on diet [56,57,58,59], adult SHBs may have actively acquired the low generic host profile via trophallactic feeding, feeding on hive products, dead bees or debris [35]. Alternatively, but not mutually exclusive, a CHC acquisition may have also passively occurred through contact with the host colony nest environment (e.g., wax comb) [22,60,61] and their honey bee hosts, as in case of ectoparasitic mites *Varroa destructor* [62].

Despite the qualitative and quantitative differences in the chemical profile between laboratory-reared and field-sampled SHBs, all SHBs taken together showed a ten-fold lower quantity of CHCs when compared to honey bee workers. Since adult SHBs are about half the size of adult honey bee workers [63], the differences in body size alone are unlikely to explain the observed difference in the total amount of CHCs. In addition, there are differences in body shape between SHBs and honey bees, leading to differences in surface area to volume ratios. This can also influence the total amount of CHCs present [64] and might partially explain the observed differences. In any case, the low amount of CHCs taken together with the relative simplicity of the CHC profile before entering host colonies might represent an adaptation to at least partly evade the honey bees’ nestmate recognition system [10]. Indeed, laboratory-reared SHBs lacked alkenes and methyl-branched alkanes instrumental for nestmate recognition in social insects [62,63]. The more chemically neutral profile of honey bee-naive beetles might favour the first host colony intrusion after emergence and could also constitute a strategy towards exploiting a broad spectrum of host bee species. However, follow up studies are required to test whether those bee-naive beetles can more successfully invade a host colony.

Interestingly, one unidentified ester (putatively acetic acid n-octadecyl ester based on its mass spectrum) was exclusively found in both laboratory-reared and field-collected SHBs, but not in honey bee workers. This ester may, therefore, constitute an intraspecific SHB recognition cue, whose actual role for communication appears worthy of investigation.

## 5. Conclusions

The present work provides the first characterization of adult SHB chemical profiles in comparison to honey bee host workers. Our data showed that adult SHBs possess a generic honey bee host CHC profile. In the field, the SHB CHC profile was not host colony-specific, probably due to adult beetles commuting between host colonies. This suggests that SHBs do not use a finely tuned chemical mimicry to conceal their presence inside a honey bee colony. The ten-fold lower CHC profiles of field-collected adult SHB compared to honey bee workers might, nevertheless, constitute an adaptation to at least partly evade nestmate recognition.

## Figures and Tables

**Table 1:** Chemical compounds detected in one-week-old laboratory-reared (lab) small hive beetles (SHB), *Aethina tumida*, field-collected SHBs and honey bee, *Apis mellifera*, host workers, and their ng/μL average amount (Mean) and standard deviation (SD) in the cuticular mixture. The results of the Kruskal–Wallis analysis and Wilcoxon post hoc tests are also shown (n.s.—not significant). Significant differences between groups (*p* < 0.05) are indicated with bold *p*-values (*—compounds not present in laboratory-reared SHBs; **—compounds not present in field-collected SHBs; ***—compounds not present in honey bee workers). Based on its mass spectrum, the unidentified ester found only in SHBs was putatively identified as an acetic acid octadecyl ester. The loadings of Partial Least Square Discriminant Analysis (PLSDA) are also shown.

Compound	Lab SHB Mean + SD	Field SHB Mean + SD	Honey Bees Mean + SD	χ^2^	*p*	Lab SHB vs. Field SHB	Lab SHB vs. Honey Bees	Field SHB vs. Honey Bees	Loadings PLSDA1	Loadings PLSDA2
C_19:1_ *^,^**	0 ± 0	0 ± 0	0.26 ± 0.48	17.158	**<0.001**	n.s.	**<0.001**	**<0.001**	0.138	−0.132
n-C_19_ *	0 ± 0	0.03 ± 0.24	1.99 ± 3.55	72.880	**<0.001**	n.s.	**<0.001**	**<0.001**	0.214	−0.004
C_21:1_ *	0 ± 0	0.04 ± 0.19	0.31 ± 0.38	26.821	**<0.001**	**0.012**	**<0.001**	**<0.001**	−0.186	−0.088
n-C_21_ *	0 ± 0	0.94 ± 0.61	10.01 ± 8.41	22.086	**<0.001**	**<0.001**	**<0.001**	n.s.	0.135	−0.052
C_22:1_	0.32 ± 0.73	0.33 ± 0.56	0.11 ± 0.24	1.544	0.492	n.s.	n.s.	n.s.	0.101	0.211
n-C_22_ *	0 ± 0	0.29 ± 0.56	3.15 ± 2.87	40.414	**<0.001**	n.s.	**<0.001**	**<0.001**	−0.047	−0.011
Unidentified ester ***	0.33 ± 0.55	0.42 ± 0.55	0 ± 0	22.007	**<0.001**	n.s.	**<0.001**	**<0.001**	0.158	0.108
C_23:1a_	2.16 ± 0.008	10.02 ± 6.21	14.99 ± 23.26	19.498	**<0.001**	n.s	**0.046**	**<0.0001**	−0.116	0.031
C_23:1b_ *	0 ± 0	0.70 ± 0.61	2.04 ± 2.86	14.413	**0.001**	**0.003**	**<0.001**	n.s.	−0.187	−0.052
n-C_23_	2.23 ± 1.36	10.74 ± 5.34	196.32 ± 295.13	0.371	0.856	n.s.	n.s.	n.s.	−0.118	0.117
C_24:1a_ *	0 ± 0	0.02 ± 0.11	0.32 ± 1.12	8.203	**0.021**	n.s.	n.s.	**0.032**	0.004	0.212
C_24:1b_	0.08 ± 0.25	0.17 ± 0.35	1.05 ± 1.94	11.404	**0.005**	n.s.	**0.046**	**0.012**	−0.013	0.086
n-C_24_ *	0 ± 0	1.10 ± 0.55	14.67 ± 12.86	23.262	**<0.001**	**<0.001**	**0.001**	n.s.	0.075	−0.093
C_25:2_ *	0 ± 0	0.08 ± 0.35	1.65 ± 5.47	23.887	**<0.001**	n.s.	**0.015**	**<0.001**	0.076	0.033
C_25:1a_	9.97 ± 8.12	17.12 ± 9.38	44.79 ± 69.01	29.544	**<0.001**	**0.015**	**<0.001**	**<0.001**	0.054	0.242
C_25:1b_	0.49 ± 0.69	1.66 ± 1.09	7.07 ± 13.19	7.711	**0.026**	n.s.	n.s.	**0.05**	0.101	−0.026
n-C_25_	4.80 ± 3.28	16.94 ± 7.54	373.49 ± 482.33	0.259	0.892	n.s.	n.s.	n.s.	−0.145	−0.006
meC_25_	0.90 ± 1.57	0.58 ± 0.72	8.21 ± 5.43	15.768	**0.001**	n.s.	**0.044**	**<0.001**	−0.031	0.224
C_26:1a_	0.09 ± 0.30	1.67 ± 1.48	0.94 ± 1.66	20.703	**<0.001**	**0.005**	**0.005**	**<0.001**	0.013	−0.007
C_26:1b_ *	0 ± 0	0.22 ± 0.53	0.33 ± 0.53	7.724	**0.026**	n.s.	**0.047**	n.s.	0.123	0.026
n-C_26_ *	0 ± 0	0.21 ± 0.45	18.38 ± 14.564	51.920	**<0.001**	n.s.	**<0.001**	**<0.001**	−0.062	0.206
meC_26a_	1.19 ± 0.49	1.41 ± 0.79	0.87 ± 1.35	41.729	**<0.001**	**0.041**	**<0.001**	**<0.001**	0.043	0.046
meC_26b_ *	0 ± 0	0.57 ± 0.83	0.48 ± 1.36	5.428	0.077	n.s.	n.s.	n.s.	0.171	0.070
C_27:1a_	1.89 ± 1.64	4.94 ± 2.45	28.30 ± 42.73	9.322	**0.013**	n.s.	n.s.	**0.007**	−0.140	−0.092
C_27:1b_	0.26 ± 0.58	0.47 ± 2.49	9.18 ± 15.63	43.211	**<0.001**	n.s.	**0.011**	**<0.001**	−0.001	0.067
n-C_27_	4.73 ± 3.81	14.88 ± 9.64	501.33 ± 341.54	0.073	0.964	n.s.	n.s.	n.s.	−0.085	0.100
meC_27a_	2.25 ± 2.54	0.04 ± 0.19	0.02 ± 0.16	28.048	**<0.001**	**<0.001**	**<0.001**	n.s.	0.153	0.010
meC_27b_	0.87 ± 1.76	1.10 ± 0.77	23.05 ± 27.22	6.683	**0.042**	n.s.	**0.019**	n.s.	0.003	−0.018
C_28:1a_	0.04 ± 0.22	0.38 ± 0.64	1.48 ± 2.70	17.695	**<0.001**	**0.010**	n.s.	**<0.001**	−0.078	−0.210
C_28:1b_ *	0 ± 0	0.33 ± 0.46	0.84 ± 1.04	7.487	**0.029**	n.s.	**0.008**	n.s.	0.047	0.148
n-C_28_	0.45 ± 0.62	1.03 ± 0.87	12.65 ± 8.86	4.377	0.128	n.s.	n.s.	n.s.	0.099	−0.184
meC_28_ **	0.20 ± 0.44	0 ± 0	1.63 ± 2.61	35.014	**<0.001**	**0.002**	n.s.	**<0.001**	0.036	0.092
C_29:2a_ *	0 ± 0	0.44 ± 0.61	1.22 ± 2.69	6.402	**0.048**	**<0.05**	n.s.	n.s.	0.072	0.142
C_29:2b_	0.08 ± 0.25	0.01 ± 0.10	0.06 ± 0.31	1.764	0.413	n.s.	n.s.	n.s.	0.131	−0.140
C_29:1a_	1.55 ± 1.21	2.33 ± 1.62	9.91 ± 18.72	37.454	**<0.001**	n.s.	**<0.001**	**<0.001**	−0.018	0.140
C_29:1b_ *	0 ± 0	0.26 ± 1.71	20.66 ± 20.56	55.416	**<0.001**	n.s.	**<0.001**	**<0.001**	0.004	−0.085
n-C_29_	12.42 ±6.83	22.11 ± 15.45	294.39 ± 204.54	8.662	**0.017**	n.s.	**<0.05**	n.s.	−0.175	0.096
meC_29a_	0.07 ± 0.22	0.69 ± 0.68	18.15 ± 24.33	15.965	**0.001**	**0.029**	**<0.001**	**0.036**	0.201	−0.039
me-C_29b_	0.19 ± 0.42	0.01 ± 0.11	2.22 ± 3.55	25.779	**<0.001**	**0.030**	n.s.	**<0.001**	−0.061	−0.007
C_30:1_ *	0 ± 0	0.07 ± 0.33	2.63 ± 2.62	54.909	**<0.001**	n.s.	**<0.001**	**<0.001**	0.108	0.137
n-C_30_	0.13 ± 0.41	0.22 ± 0.51	7.05 ± 6.33	47.351	**<0.001**	n.s.	**<0.001**	**<0.001**	0.122	−0.141
meC_30_ *^,^**	0 ± 0	0 ± 0	0.49 ± 0.93	17.158	**<0.001**	n.s.	**<0.001**	**<0.001**	0.198	−0.031
C_31:2a_ *^,^**	0 ± 0	0 ± 0	0.31 ± 0.94	8.694	**0.017**	n.s.	**0.022**	**0.014**	0.176	0.035
C_31:2b_ *	0 ± 0	0.04 ± 0.27	4.26 ± 4.63	55.987	**<0.001**	n.s.	**<0.001**	**<0.001**	0.114	−0.075
C_31:1a_ *	0 ± 0	4.69 ± 7.10	89.27 ± 70.97	24.395	**<0.001**	**<0.001**	**<0.001**	n.s.	0.092	−0.066
C_31:1b_ *	0 ± 0	4.10 ± 6.16	87.23 ± 71.41	25.265	**<0.001**	**<0.001**	**<0.001**	n.s.	0.197	−0.043
n-C_31_	1.21 ± 0.92	4.74 ± 7.48	196.39 ± 158.69	3.335	0.211	n.s.	n.s.	n.s.	0.083	0.314
meC_31a_ **	0.08 ± 0.25	0 ± 0	6.30 ± 9.13	64.691	**<0.001**	**0.020**	**<0.001**	**<0.001**	0.068	0.292
meC_31b_ *^,^**	0 ± 0	0 ± 0	1.52 ± 1.74	53.756	**<0.001**	n.s.	**<0.001**	**<0.001**	0.012	0.140
C_32:1_ *	0 ± 0	0.11 ± 0.59	8.07 ± 6.43	69.219	**<0.001**	n.s.	**<0.001**	**<0.001**	0.202	−0.045
C_33:2_	3.96 ± 3.34	3.49 ± 3.12	22.24 ± 23.78	3.129	0.230	n.s.	n.s.	n.s.	0.192	−0.070
C_33:1_	0.29 ± 0.94	9.73 ± 15.77	263.50 ± 190.31	24.503	**<0.001**	**<0.001**	**<0.001**	n.s.	0.207	−0.002
n-C_33_ *	0 ± 0	0.01 ± 0.11	14.76 ± 26.10	27.807	**<0.001**	n.s.	**<0.001**	**<0.001**	−0.012	−0.002
meC_33_ *	0 ± 0	0.53 ± 1.85	1.16 ± 2.26	9.590	**0.008**	n.s.	n.s.	**0.027**	0.077	0.309
C_35:2_ *	0 ± 0	0.03 ± 0.24	2.30 ± 2.97	35.511	**<0.001**	n.s.	**0.007**	**<0.001**	0.167	−0.125
C_35:1a_ *	0 ± 0	0.04 ± 0.30	4.18 ± 4.80	43.226	**<0.001**	n.s.	**0.002**	**<0.001**	0.083	−0.018
C_35:1b_ *	0 ± 0	0 ± 0	3.40 ± 7.35	28.375	**<0.001**	n.s.	**0.019**	**<0.001**	0.173	−0.098
Oleic acid ester 1 *	0 ± 0	0.65 ± 2.27	19.59 ± 18.43	40.351	**<0.001**	n.s.	**0.001**	**<0.001**	0.179	−0.057
Oleic acid ester 2 *	0 ± 0	0.85 ± 4.98	46.01 ± 47.44	53.180	**<0.001**	n.s.	**<0.001**	**<0.001**	0.106	0.053
Oleic acid ester 3	29.01 ± 19.68	9.55 ± 8.87	7.95 ± 30.33	43.506	**<0.001**	n.s.	**<0.001**	**<0.001**	−0.012	−0.107
Oleic acid ester 4	9.58 ± 7.28	3.44 ± 3.99	3.91 ± 20.67	39.310	**<0.001**	n.s.	**<0.001**	**<0.001**	0.158	0.009

## Data Availability

The complete raw data will be deposited at the Dryad repository upon acceptance for publication.
